# Chemistry of enzymatic browning in longan fruit as a function of pericarp pH and dehydration and its prevention by essential oil, an alternative approach to SO_2_ fumigation

**DOI:** 10.7717/peerj.11539

**Published:** 2021-06-14

**Authors:** Muhammad Rafiullah Khan, Chongxing Huang, Yasser Durrani, Ali Muhammad

**Affiliations:** 1 School of Light Industry and Food Engineering, Guangxi University, Nanning, China; 2 Department of Food Science and Technology, The University of Agriculture, Peshawar, Pakistan

**Keywords:** Longan, Enzymatic browning, Antimicrobial, Essential oil, Thymol, Fruit

## Abstract

**Background:**

Longan fruit is a rich source of bioactive compounds; however, enzymatic browning of pericarp and microbial decay have limited its postharvest life. SO_2_ has widely been used to overcome these limitations; however, due to safety and regulatory concerns, alternative means should be identified. In this study, antioxidant and antimicrobial properties of thymol (TH) essential oil were investigated against the enzymatic browning and decay of longan fruit.

**Methods:**

Fruits were coated with TH (4%) for 5 min, sealed in polyethylene (PE) packages and stored at 4 °C for 42 d. Fruits immersed in distilled water (DW) and stored in PE were used as control.

**Results:**

TH extended the postharvest life of longan to 42 d than 28 d in DW. TH residues decreased from 142 to 11.17 mg kg^–1^, while no residues were found at day 42. TH significantly (*P* ≤ 0.05) reduced the respiration rate, inhibited polyphenol oxidase (PPO) and peroxidase (POD) enzyme activities, sustained high phenols/flavonoids and prevented pericarp browning (BI) than DW. TH also effectively (*P* ≤ 0.05) maintained the color values, firmness of peel and aril, total soluble solids (TSS), titratable acidity (TA), inhibited decay incidence (DI) and resulted in lower ethanol content than DW. BI as a function of pericarp pH was highly correlated; pH and BI (*r* = 0. 97), with PPO (*r* = 0.93) and with water loss (*r* = 0.99). A high coefficient of correlation of BI was found with the pericarp pH, enzymes, phenolic, water loss and decay incidence with ethanol. TH could be the best alternative to SO_2_ and other synthetic preservatives.

## Introduction

Longan (*Dimocarpus longan* Lour.) fruit is a rich source of vitamins and minerals and bioactive compounds which exhibit antibacterial, antiviral, antioxidant, anti-inflammatory and anti-carcinogenic properties, anti-tyrosinase activities, as well as memory-enhancing effects and, have been used as bioactive constituents in traditional Asian medicines for different treatments (Fu et al., 2011; Jiang et al., 2013; Lal et al., 2020; Prasad, Neha & Lal, 2017; Shahrajabian, Sun & Cheng, 2019; Wall, 2006; Yang et al., 2011; Zheng et al., 2009; Zhu et al., 2019). Short postharvest life of longan fruit due to the pericarp browning and postharvest decay had limited its storage and transportation to the long distant and international markets. Sulfur dioxide fumigation has commonly been used to overcome these limitations and to extend the longan fruit’s storage life. However, due to increased safety concerns and regulatory issues associated with sulfur dioxide, its uses have been banned (European Parliament, 1995).

Various attempts had been made to prevent pericarp browning and postharvest decay of longan fruit. For example, *Chomkitichai et al. (2014)* fumigated longan fruits with chlorine dioxide which enhanced the antioxidant defence system of the postharvested longan fruits. *Lin et al. (2015)* studied the inhibitory effects of propyl gallate on browning and its relationship to active oxygen metabolism in the pericarp of harvested longan fruit. *Lin et al. (2017)* alleviated the pericarp browning of harvested longan fruit by using propyl gallate to modulate the metabolisms of respiration rate and energy. *Lin et al. (2019)* alleviated the pulp breakdown of harvested longan fruit by using chitosan to suppress the disassembly of cell wall polysaccharides. *Joradol et al. (2019)* treated longan fruits with SO_2_, ClO_2_ or their combination which reduced the pericarp browning and maintained high fruit quality. These fumigations induced early transient H_2_O_2_ generation by the nicotinamide adenine dinucleotide phosphate oxidase (NOX) and superoxide dismutase (SOD) system that triggers the antioxidant response.

Natural means have widely been studied as alternatives to sulfur dioxide treatments. Essential oils are plant-based extracts containing several volatile compounds having antioxidant, antimicrobial properties and diverse biological activities (Akbari, Gholami & Ghobad, 2019; Das et al., 2021; Hemeg et al., 2020). Thymol, menthol and eugenol were shown to reduce decay and increase total phenol, total anthocyanin and oxygen radical absorbance capacity (ORAC) in strawberries (Wang et al., 2007). Carboxymethylcellulose coating associated with essential oil had increased the shelf life of papaya with good quality attributes (Zillo et al., 2018). Cinnamaldehyde combined with chitosan coating preserved the quality and extended the postharvest life of navel oranges (Gao et al., 2018). Edible coating combined with essential oil, prevented microbial growth and extended the shelf life of fresh-cut melons (Yousuf, Srivastava & Ahmad, 2020) and papaya (Batista et al., 2020). Alginate coating enriched with thyme essential oil extended the shelf life of pistachio (Hashemi et al., 2020). Our research team (Haohe et al., 2020) prepared microcapsules from oregano essential oil and *β*-cyclodextrin and extended the shelf life of fresh-cut purple yam.

Longan is an economically important fruit, but limited information is available to prevent its enzymatic browning and microbial decay by natural means. Therefore, this study aimed to evaluate the antioxidant and antimicrobial activity of thymol against the longan fruit. pH playing an important role in the enzymatic browning reactions; therefore, in this study, along with the enzymes, their phenolic substrates and interactions with pH were also evaluated. Other physico-chemical quality tests were conducted to monitor the overall market changes. Furthermore, although essential oils are generally recognized as safe (GRAS) compounds, their residues should not exceed 50 mg kg^−^^1^. Therefore, TH residues were also determined to provide safe levels of essential oils in foods and fresh produce industries.

## Materials & Methods

### Plant materials and treatments

Longan (*Dimocarpus longan* Lour.) fruits cv. Daw was obtained from the nearby commercial orchard. Fruits with no defects were sorted for uniform color and size and surface sterilized with sodium hypochlorite (NaClO) (0.1%) for 1 min. Thymol (>99% FCC, Sigma-Aldrich) solution was prepared by dispersing 40.0 g of thymol in 200 mL of distilled water, to which 10 mL of absolute ethanol was added and the volume was adjusted to 1 L by distilled water. Fruits (about 500 g) were treated with TH for 5 min, air-dried by a fan for 40 min in room temperature and packed in low-density polyethylene (PE) bags having a thickness of 55 ± 2 µm. Fruit dipped in distilled water, dried and packed in PE bags were considered as control. Each experiment was conducted in triplicate. All packages were stored at 4 °C and the quality parameters were evaluated every 7 d.

### Respiration rate measurement

Respiration rate was measured in a closed system at 4 °C. Approximately 250 g of fruits were kept in a 750 mL jar and hermetically sealed for 1 h. Gas concentration in the jar was measured by withdrawing five mL of headspace gas using a hypodermic syringe and injected into the gas chromatograph (model 6890N, Agilent Technologies, USA) equipped with a thermal conductivity detector (oven temperature 60 °C; detector temperature 200 °C) and HayeSep Q 100/120 packed column (Alltech Associates, Inc.) according to the method of *Boonruang et al. (2012).* Respiration rate was calculated as mL CO_2_ kg^−^^1^h^−^^1^ on a fresh weight base. In each treatment, the respiration rate was measured in three replicates.

### Antibrowning activities of thymol

Pericarp browning was determined according to Jiang & Li (2001) using 45 to 55 fruits (500 g) in each package. From each treatment, three packages were used on each respective day of analysis.

Polyphenoloxidase (PPO) and peroxidase (POD) enzymes were extracted according *to Duan et al. (2007)* using pericarps of 10 fruits. Activities of these enzymes were determined by the methods of Jiang (1999) and Zhang et al. (2005), respectively. Phenolic and flavonoid contents were extracted from the pericarps of 10 fruits and analyzed by the method of Marinova, Ribarov & Atanassova (2005) and Dewanto et al. (2002), respectively. Enzymes and phenolic experiments were done in six replicates.

### Pericarp color, pericarp pH, weight loss, firmness, total soluble solids (TSS) and titratable acidity (TA)

Outer pericarp color (L*, a*, b*) was measured using Minolta CR-310 colorimeter (Minolta, Tokyo, Japan), taking 3 fruits from each replicate and their average was reported. Chroma (C*) and hue (*h*^∘^**) were calculated. The pH of the pericarp was determined using a pH meter. Weight loss was determined by weighing the fruit on day 0 and every 7 d and the results were expressed in percentage. The firmnesses of pericarp and flesh were measured using a Testometric (MICRO 350, England) in a compression mode. A two mm diameter plunger was used to puncture the fruit to a depth of five mm at speed of 20 mm min^−1^ at the equatorial region. The maximum force to penetrate the fruit was recorded Newton (N). A total of 3 fruits were used for each test. For TSS and TA, the flesh of about 10 fruits was ground, filtered through muslin cloth and clear juice was used for TSS and TA according to Duan et al. (2007), while the remaining juice was stored for ethanol measurement. TSS and TA tests were conducted in triplicate.

### Decay incidence and ethanol content

Any fruit with visible microbial growth was considered decayed and the results were expressed in percentage. For ethanol content 10 mL of juice was sealed in 20 mL vials and stored at −20 °C. Ethanol content were then determined according to the method of *Larsen & Watkins (1995)* using a gas chromatograph (Model 6820, Agilent Technologies, USA) equipped with a flame ionization detector (FID) and DB −5 column (30 m ×0.250 mm, 0.25 mic). Results are the mean of three replicate ± standard deviation and expressed in mol L^−^^1^ of juice.

### Determination of thymol residues in longan pericarp

Thymol residues were measured by the solubility method of *Bermejo et al. (2015)* with slight modification. Pericarps of 10 fruit was ground and sieved using mesh no. 30 with an aperture 600 µm (Retsch, D5657, Germany). Fine powder (1.0 g) of the ground pericarp was taken in a test tube, to which 10 mL of absolute ethanol was added and sealed. The test tubes were put in a water bath with a shaker (to agitate the samples) at 40 °C for 24 h. After equilibrium, the samples were kept at room temperature for 48 h for a complete phase separation. A clear sample of five mL was pipetted in the amber bottles and 0.2 µL of the extract was taken by a glass syringe and injected into a gas chromatograph (HP −6820, Guangzhou, China) equipped with a flame ionization detector (FID) and DB −5 column (30m ×0.250 mm, 0.25mic). Helium at a rate of two mL min^−1^ was used as a carrier gas. Experimental conditions were; 80–100 °C oven temperature, 250 °C injector temperature, 280 °C detector temperature. The experiment was conducted in triplicate.

### Statistical analysis

Data were subjected to analysis of variance (ANOVA) using Statistix software. The least significant difference tests were performed to determine the significant difference (*P* ≤ 0.05) among the treatments. The correlation analysis was carried out in Microsoft Excel 2016.

## Results

**Figure 1:**
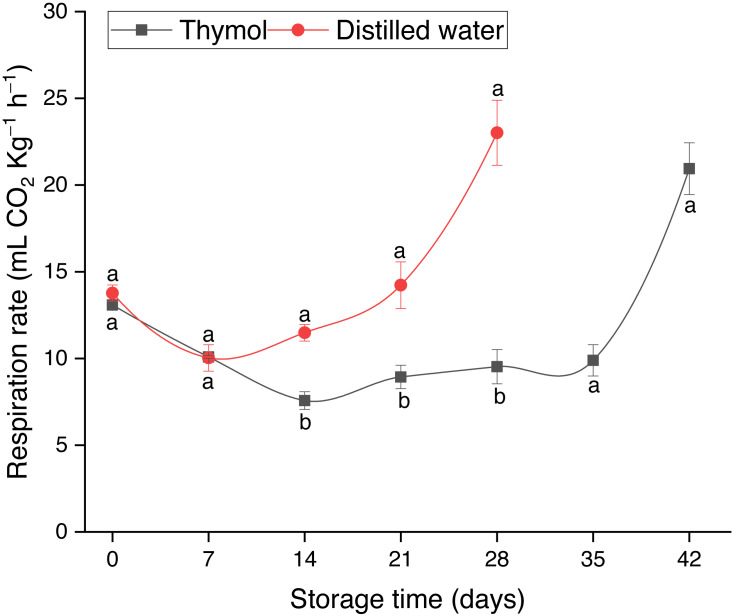
Respiration of longan treated with TH and stored at 4 °C. Each data point represents a mean (*n* = 3) ± SD.

**Figure 2:**
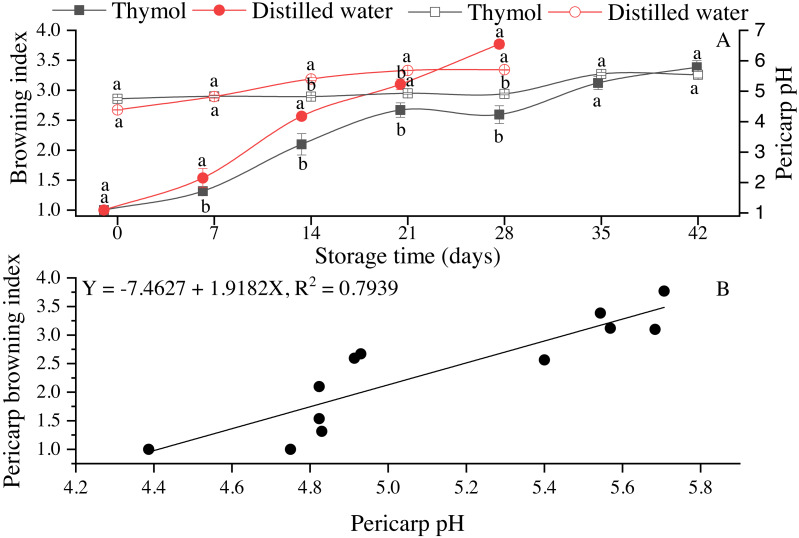
(A) Pericarp browning index (closed symbols) and pericarp pH values (open symbols). (B) Pericarp browning as a function of pericarp pH in longan fruit treated with TH and stored at 4 °C. Each data point represents a mean (*n* = 3) ± SD. Browning scale, 1 = 0% (no browning), 2 = 1–10% (slight browning), 3 = >10–25% (moderate browning), 4 = 26–50% (severe browning) and 5 = >50%.

**Figure 3:**
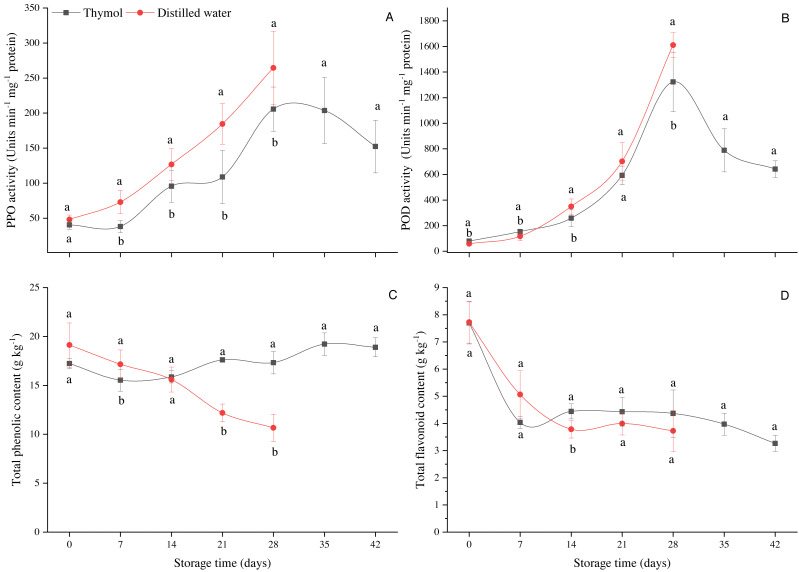
(A) Changes in PPO activity, (B) POD activity, (C) total phenolic content and (D) total flavonoid content in longan treated with TH and stored at 4 °C. Each data point represents a mean ± SD (*n* = 6).

Respiration rate slightly decreased up to day 14 in both treatments and then increased being significantly higher (*P* ≤ 0.05) in DW than TH (Fig. 1). Pericarp browning (BI) gradually enhanced in both treatments (Fig. 2A); however, in the control treatment, BI was significantly high due to which the fruit storage was ended on day 28. The pH values in the TH treated fruit ranged from 4.75–5.54, while in DW, pH values were 4.38 to 5.71 (Fig. 2A). Pericarp browning as a function of pericarp pH is presented in Fig. 2B. Thymol effectively inhibited the PPO activity as can be seen that changes in PPO activity were very low (*P* ≤ 0.05) in the TH treated fruit than the control (DW) (Fig. 3A). PPO activity continuously increased, which resulted in a brown pericarp in DW treatment. Peroxidase (POD) activity first slightly increased in both treatments and then gradually declined with storage time (Fig. 3B). TPC and TFC in longan pericarp slightly decreased in TH treated fruit and then gradually increased. However, in non-treated fruit, TPC and TFC were continuously decreased (*P* ≤ 0.05) (Figs. 3C & 3D). This shows that PPO had oxidized phenolic compounds as high PPO activity was found in DW (Fig. 3A), which resulted in a high BI (Fig. 2A).

**Figure 4:**
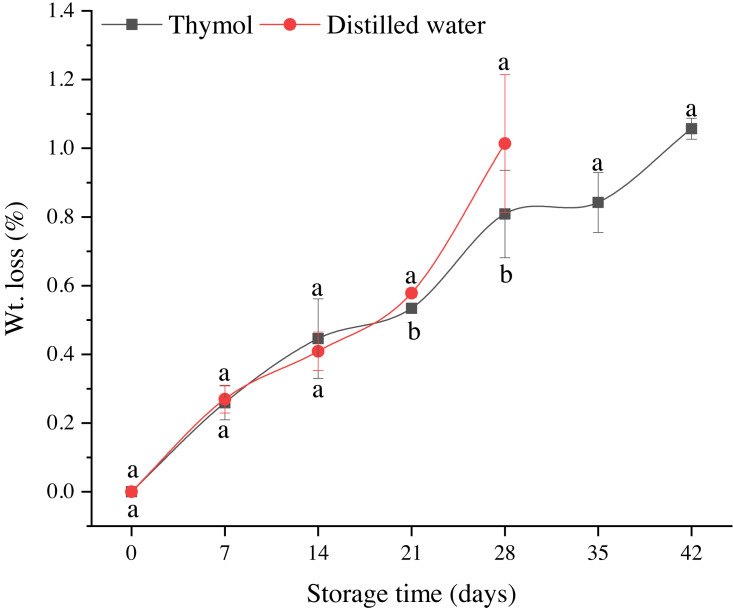
Weight loss of longan treated with TH and stored at 4 °C. Each data point represents a mean ± SD (*n* = 3).

Weight loss was non-significantly different in both treatments until day 14 whereas then significantly higher in DW than TH. However, there was an increase in the weight loss with storage time (Fig. 4). Weight loss resulted in the highest BI, which can be seen from its highly positive correlation with BI (*r* =0.99) and negative correlation with L* (*r* = −0.88). Regarding the color values, lightness (L*) gradually decreased in both treatments with the storage time. The highest decrease was found in the control treatment. TH treated fruit had high L* values as compared to DW. Redness (a*) values increased in both treatments, however, there was no significant difference except on day 28 (Table 1). Yellowness (b*) and color intensity (chroma; C*) of longan fruit decreased in both treatments with storage time (Table 1). Among these treatments, TH treated fruits had higher b* and C* values throughout the storage period than DW except at days 7 and 14. Similarly, TH also maintained the hue angles (*h*^∘^**) of longan pericarp better than DW (Table 1). The firmness of the peel and flesh of longan fruit gradually declined with storage time (Table 2). No significant difference was found among the treatments in the initial storage time; however, firmness decreased (*P* ≤ 0.05) in control treatment at the end, probably due to the high decay incidence. TSS and TA of longan flesh decreased with storage (Table 2). No significant difference was observed in TH and control treatment.

**Figure 5:**
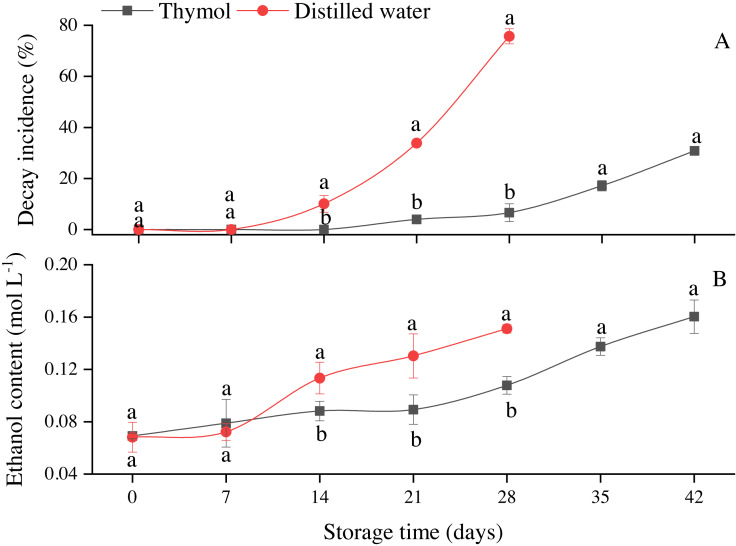
(A) Decay incidence and (B) ethanol content in longan treated with TH and stored at 4 °C. Each data point represents a mean ± SD (*n* = 3).

Thymol is an effective antimicrobial agent and significantly (*P* ≤ 0.05) inhibited DI as compared to DW (Fig. 5A). On the other hand, ethanol concentration in longan flesh gradually increased in both treatments (Fig. 5B). In general, ethanol increased with storage time and highest at the end of the storage. The highest (*P* ≤ 0.05) ethanol contents were observed in control on day 14 and onward.

**Figure 6:**
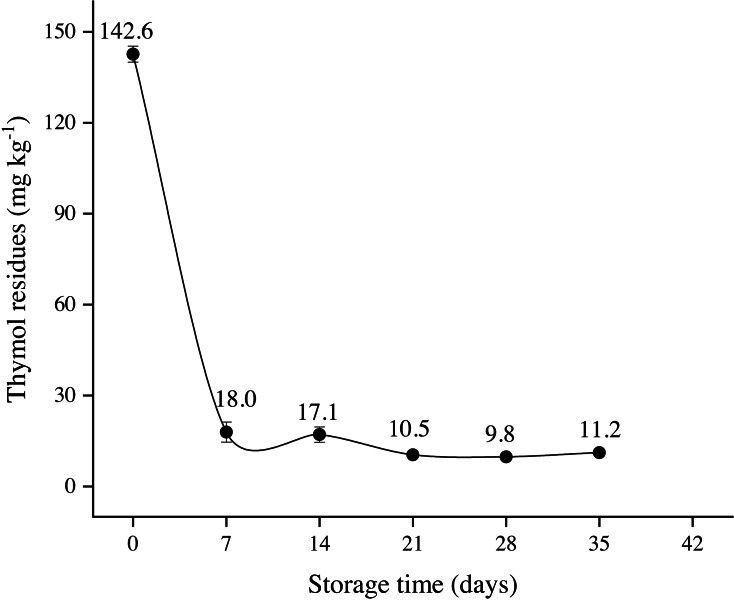
Thymol residues in longan pericarp treated with TH and stored at 4 °C. Each data point represents a mean ± SD (*n* = 3).

TH residues in longan pericarp continuously decreased. At day 0, TH residues were significantly higher (*P* ≤ 0.05), then reduced with storage time (Fig. 6). TH residues decreased from 142.63 mg kg^−^^1^ on day 0 to 11.17 mg kg^−^^1^ on day 35, while no TH residues were detected on day 42.

## Discussion

Reducing the metabolic activity of the post harvested fresh produce is an effective way to extend their postharvest life. As can be seen in Fig. 1 that TH in combination with low temperature resulted in a significant reduction in the respiration rate of longan fruit due to lowering the metabolic activity; it was probably induced by TH as previously reported in apricots (Wu et al., 2014). Another reason for the lower respiration rate in the TH treated fruit could be attributed to the formation of the coated layer, which had modified the endogenous O_2_ and CO_2_ and reduced the O_2_ supply (Wang, Qian & Ding, 2018). It is noteworthy to mention that longan fruits are non–climacteric and there is no sudden increase in the respiration rate after harvest. Therefore, this increase in the respiration rate could be associated with the decay incidence *(Jiang et al., 2002)*, as high decay incidence was observed during this period. An increase in the respiration rate associated with the decay incidence was previously found in longan by *Jiang & Li (2001)* when fruits were coated with chitosan at different concentrations (0.5, 1.0 and 2.0%).

**Table 1:** Color values of longan fruit treated with thymol and stored at 4 ° C.

Parameters	Treatments	Storage time (days)						
		0	7	14	21	28	35	42
L*	TH	62.6 ± 0.7^a^	58.5 ± 1.0^a^	57.1 ± 1.0^a^	50.8 ± 1.4^a^	46.4 ± 1.4^a^	46.4 ± 1.1^a^	42.4 ± 1.8^a^
	DW	50.9 ± 1.9^b^	57.3 ± 3.5^a^	57.2 ± 0.4^a^	43.5 ± 1.3^b^	40.8 ± 1.6^b^	–	–
a*	TH	3.7 ± 0.3^a^	3.8 ± 0.2^a^	4.2 ± 0.2^a^	4.2 ± 0.0^a^	4.5 ± 0.1^a^	4.4 ± 0.4^a^	4.6 ± 0.3^a^
	DW	2.4 ± 0.2^b^	3.6 ± 0.2^a^	4.5 ± 0.1^a^	4.2 ± 0.3^a^	3.5 ± 0.2^b^	–	–
b*	TH	22.5 ± 0.3^a^	27.2 ± 0.2^a^	26.5 ± 0.5^a^	23.4 ± 0.1^a^	21.4 ± 1.8^a^	20.8 ± 1.7^a^	18.6 ± 1.4^a^
	DW	17.8 ± 0.9^b^	26.5 ± 2.0^a^	25.8 ± 2.0^a^	18.6 ± 1.1^b^	17.6 ± 0.7^b^	–	–
C*	TH	22.8 ± 0.3^a^	27.4 ± 0.3^a^	26.8 ± 0.5^a^	23.8 ± 0.2^a^	21.9 ± 1.7^a^	21.3 ± 1.8^a^	19.2 ± 1.4^a^
	DW	18.0 ± 0.8^b^	26.8 ± 1.9^a^	26.1 ± 2.0^a^	19.2 ± 1.1^b^	18.0 ± 0.6^b^	–	–
*h*^∘^**	TH	80.6 ± 0.1^a^	82.0 ± 1.1^a^	81.3 ± 0.4^a^	79.8 ± 0.5^a^	77.9 ± 1.1^a^	77.9 ± 0.7^a^	75.9 ± 2.4^a^
	DW	82.6 ± 1.0^a^	82.3 ± 0.9^a^	80.2 ± 0.2^a^	77.5 ± 0.23^a^	78.5 ± 1.5^a^	–	–

**Notes.**

Each data point represents a mean ± standard deviation (*n* = 3). Different small letters within each column represent a significant difference (*P* ≤ 0.05).

TH Thmol DW Distilled water

**Table 2:** Firmness (N), total soluble solids (TSS, %) and titratable acidity (TA, %) of longan fruit treated with TH and stored at 4 °C.

Parameters	Treatments	Storage time (days)						
		0	7	14	21	28	35	42
Firmness (pericarp)	TH	18.3 ± 1.2^a^	14.4 ± 1.8^a^	14.1 ± 3.6^a^	14.5 ± 0.2^a^	13.8 ± 1.2^a^	8.7 ± 1.5^a^	8.4 ± 2.7^a^
	DW	17.3 ± 2.3^a^	14.0 ± 0.4^a^	13.1 ± 2.0^a^	9.5 ± 0.8^b^	6.4 ± 1.3^b^	–	–
Firmness (flesh)	TH	2.6 ± 0.9^a^	2.3 ± 0.2^a^	2.1 ± 0.6^a^	2.0 ± 0.4^a^	1.8 ± 0.3^a^	1.8 ± 0.4^a^	2.2 ± 0.9^a^
	DW	2.3 ± 0.6^a^	2.1 ± 0.6^a^	1.8 ± 0.4^a^	1.9 ± 0.3^a^	0.7 ± 0.5^b^	–	–
TSS	TH	19.7 ± 0.5^a^	18.7 ± 0.5^a^	18.7 ± 0.2^a^	18.1 ± 0.3^a^	18.0 ± 0.0^a^	17.9 ± 0.1^a^	17.2 ± 0.2^a^
	DW	19.4 ± 0.2^a^	18.5 ± 0.5^a^	18.5 ± 0.5^a^	18.1 ± 0.1^a^	17.4 ± 0.3^b^	–	–
TA	TH	0.21 ± 0.0^b^	0.2 ± 0.0^a^	0.2 ± 0.0^a^	0.2 ± 0.0^a^	0.2 ± 0.0^a^	0.2 ± 0.0^a^	0.2 ± 0.0^a^
	DW	0.2 ± 0.0^a^	0.2 ± 0.0^a^	0.2 ± 0.0^a^	0.2 ± 0.0^a^	0.2 ± 0.0^a^	–	–

**Notes.**

Each data point represents a mean ± standard deviation (*n* = 3). Different small letters within each column represent a significant difference (*P* ≤ 0.05).

TH Thymol DW Distilled water

Pericarp browning (BI) is one of the critical limiting factors that limit the fruit postharvest life and quality. Enzymatic browning phenomenon currently gaining much attention from food industries for its safe prevention due to substantial economic loss. TH treated fruit have comparatively low BI until day 42, which indicated that TH could prevent phenolic compounds’ oxidation due to its antioxidant activities (Akbari, Gholami & Ghobad, 2019).

**Figure 7:**
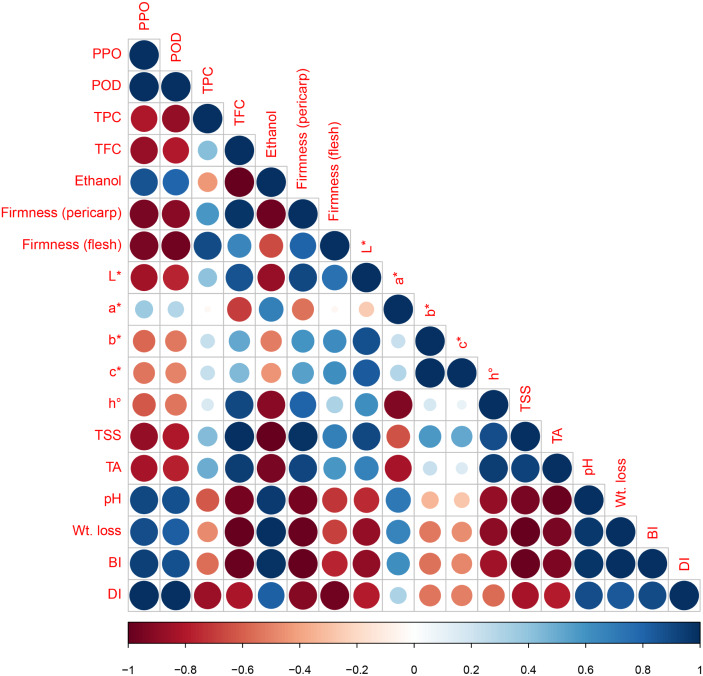
Pearson coefficient of correlation in different quality parameters in longan fruit stored at 4 °C.

pH is one of the main factors in the enzyme system. BI of longan fruit is mainly associated with PPO and POD activities. Therefore, the pH of the pericarp and its association with the BI and enzymes was determined. In this study, upon the correlation coefficient, factors such as pH, PPO and phenolic substrates being involved in the enzymatic browning reaction, were highly correlated as high positive correlations were found between the pericarp pH and BI (*r* =0.97), with phenolic compounds (*r* = −0.61) and with PPO (*r* =0.93). In Pearson correlation coefficients analysis, different quality parameters in longan fruit during the storage were highly correlated (Fig. 7). This correlation can be seen in their respective figures, where high PPO activity (Fig. 3A) and high BI (Fig. 2A) with low total phenolic contents (Fig. 3C). Lower pericarp pH in association with lower BI in longan cv. Daw was also observed by Apai (2010) when fruits were dipped for 20 min in hydrochloric acid (HCl), stored at 3 °C for 60 d.

It has widely been accepted that oxidation of the phenolic substrates by PPO is one of the major causes of browning of fresh fruits and vegetables. POD is an oxidoreductase enzyme that plays a vital role in antioxidant defense during the fruit ripening/senescence process, retards enzymatic browning where POD decomposes H_2_O_2_ by oxidation of co-substrates such as phenolic compounds *(Chisari, Barbagallo & Spagna, 2007)*. The highest TPC and TFC in TH treatment were due to the high antioxidant characteristics of TH, which played a vital role in systemic resistance and an anti-senescence effect in harvested fruit (Alenazi et al., 2020). *Wang et al. (2007)* reported that essential oil could generate mild stress and the fruit produce additional phenolic and flavonoid content in their defensive response. Our results agree with Wang et al. (2007), who treated strawberries with thymol, eugenol and menthol and reported that total phenols, flavonoids, anthocyanins and antioxidant capacities were higher than the control (non-treated) strawberries, stored at 10 °C for 3 weeks. High total phenolic content was also found in MAP + thymol or eugenol (essential oil) in table grapes compared to control treatment *(Valero et al., 2006).* An increase in the PPO and POD activities associated with the increase in the pericarp browning and decrease in the phenolic content were also reported by *Jiang & Li (2001)* and *Duan et al. (2007)* when longan fruits were treated with chitosan and nitic acid respecivley.

Weight loss is one of the significant and primary factors contributing to the pericarp browning, probably due to the disintegration of the cell membrane where phenolic compounds come into contact with the enzymes and initiating a browning reaction. Therefore, its relation with the pericarp browning and phenolic content was studied in this work. TH treatment had an effect on the weight loss of longan fruit by lowering the dehydration process; however, in all packages, weight loss was less than 1.0%, mainly due to the water vapor accumulation within the packages, as one of the major advantages of polymeric films. Furthermore, a positive effect of TH on longan fruit can be seen on the color values of the pericarp, which suggests that the coating of longan fruit delayed the senescence, and resulted in better color maintenance than uncoated fruit. Thymol also significantly maintained the color changes (L*, a* and b*) in table grapes than control treatment (Valero et al., 2006).

Mechanical properties of fruit depend on cell wall strength, cell-to-cell adhesion, cell packing and the internal pressure or turgor of the cell. *Serrano et al. (2005)* fumigated the sweet cherries with different essential oils, stored at 1 °C for 16 d and reported that eugenol followed by thymol got the best results in controlling the loss of firmness due to the reduction in decay incidence and less susceptibility to mechanical damage. Generally, high TSS and TA contents were observed in TH treated longan than distilled water (control) which were also found in TH treated strawberries than untreated (control), stored at 10 °C for 14 d (Wang et al., 2007).

The effective (*P* ≤ 0.05) inhibition of decay incidence by TH was probably due to its hydrophobicity characteristic, which facilitated the partition of the lipids in the cell membrane, damaged membrane protein, inactivated the essential enzymes and disturbed the functionality of their genetic material (Khorshidian et al., 2018). Another possible reason could be that TH probably maintained the metabolic activity of the fruit and effectively reduced the respiration rate (Fig. 1). *Khorshidian et al. (2018)* reported that the presence of the phenolic ring and its toxicity which are related to the number of hydroxyl groups in the phenolic ring could be the reason for the antimicrobial activity of TH. Thymol also prevented microbial growth in other fruits such as cherries (Serrano et al., 2005) and table grapes (Valero et al., 2006; Valverde et al., 2005).

Throughout storage, TH resulted in a significant lowest ethanol content in longan flesh, suggesting that TH maintained the cell integrity, metabolic activity of the fruit. This was associated with its antimicrobial characteristics of TH, which effetely prevented the decay of longan fruit. High ethanol contents were probably associated with excessive DI as a high correlation coefficient (*r* =0.82) was found in DI and ethanol content. Essential oils are considered generally recognized as safe (GRAS) for food ingredients, but their concentration should not exceed 50 mg kg^−^^1^ (United States Government Publishing Office, 2003; section VI); hence could be used to prevent the postharvest decay of the fruit. Interestingly, in the current study, TH residues concentration in longan pericarp was far below the permissible level on day 7 and onward and effectively controlled the decay of longan fruit.

## Conclusions

Essential oils are generally recognized as safe (GRAS) compounds and currently gain much interest from researchers. Antioxidant and antimicrobial characteristics of TH were evaluated against the enzymatic browning and microbial decay of longan fruit. TH effectively inhibited enzyme activities, oxidation of the phenolic compounds, kept higher color values and thus delayed the longan pericarp browning than DW (control). TH also prevented the growth of microorganisms, maintained the overall quality and extended the shelf life of logan to 42 d than 28 d in control. TH residues in longan pericarp decreased from 142 to 11.17 mg kg^−^^1^ and no residues were detected at day 42. TH could be the best alternative to SO_2_ in food and fresh produce to preserve the overall quality of the fresh produce and food products. This approach is a relatively simple but very economical preservative method to prevent BI and DI with the longest shelf life.

##  Supplemental Information

10.7717/peerj.11539/supp-1 Supplemental Information 1 Raw data

## Additional Information and Declarations

### Data Availability

The following information was supplied regarding data availability: The raw data are available in the Supplementary File.
